# INFLUENCE OF HEALTH CARE PROFESSIONALS ON END-OF-LIFE AND PLACE OF DEATH: A LATENT CLASS ANALYSIS

**DOI:** 10.1093/geroni/igae098.0749

**Published:** 2024-12-31

**Authors:** Autumn Decker, Raven Weaver, Cory Bolkan, Brittany Cooper

**Affiliations:** Pacific University, Hillsboro, Oregon, United States; Washington State University, Pullman, Washington, United States; Washington State University, Pullman, Washington, United States; Washington State University, Pullman, Washington, United States

## Abstract

People experience end-of-life (EOL) differently due to unique lived experience and sociopolitical position. Most people prefer to die at home, and evidence suggests that healthcare providers have a role to play in fostering a positive EOL experience. This study aimed to understand classes of healthcare provider involvement in the EOL experience and examine the association between class membership and place of death by living alone, race, and/or geographic location. We conducted a latent class analysis with covariates and a distal outcome using Waves 9-12 (n=1290) from the last month of life interview in the National Health and Aging Trends Study. Based on fit statistics, response probabilities, and class separation, a four-class model was selected. These classes were Discordant Death (DD), Controlled Death (CD), Harmonious Death, and Comforted Death. Living alone (p <.001), being White (p =.015) or Black (p =.047), and living in the Northeast (p =.015) or Midwest (p =.039) were significantly associated with latent class membership. Those living alone and in the DD class were most likely to die in a location outside the home, Black individuals in the CD class were most likely to die in a hospital, and those living in the Northwest and in the CD class had the highest probability of dying at home. This work demonstrates that health provider factors have influence over the experiences one has at EOL, and ultimately, their place of death. Future research should explore concordance between EOL wishes and the EOL experience.

